# Introduction to the Outlines of the Principles of Differential Diagnosis, with Clinical Memoranda

**Published:** 1900-09

**Authors:** 


**Introduction to the Outlines of the Principles of Differential Diagnosis, with Clinical Memoranda.** By Fred. J. Smith, M.D. Pp. ix., 353. London: Macmillan and Co., Limited. 1899.

When one reads through this book with care, it is at once evident that a very great amount of thought and experience has been expended on it. Its chief aim seems to be the diagnosis of cases by working through the list of possibilities indicated by certain combinations of symptoms. The book is therefore full of elaborate classifications of symptoms, to each of which is attached a note on its importance in the diagnosis. There is very little to say about the subject matter; the book does not pretend to have anything new in this direction. All possible differential diagnoses are tabulated in columns, making a beginner think medicine is a finite science which merely requires a good memory to acquire.

If the book is meant for students, it is appalling in the amount of possibilities which a case may present. There are, for instance, elaborate tables on the diagnosis of various heart lesions; but by the time the symptoms are put together and fitted to the correct theoretical diagnosis, and applied to the patient, the mind is in such a muddle that it seems best to put it all on one side and start afresh. The chief fault lies in the fact that it is too theoretical and not clinical enough. It may be useful to the student as a book of principles, and may be recommended to young teachers to look through before going over a particular class of case with the clinical clerks.

